# Addition of metformin for non-small cell lung cancer patients receiving antineoplastic agents

**DOI:** 10.3389/fphar.2023.1123834

**Published:** 2023-03-10

**Authors:** Yan Wang, Yuanyuan Hu, Ting Wang, Guowei Che, Lu Li

**Affiliations:** ^1^ Department of Thoracic Surgery, West China Hospital, Sichuan University, Chengdu, China; ^2^ Department of Obstetrics and Gynecology, West China Second University Hospital of Sichuan University, Chengdu, Sichuan, China; ^3^ Lung Cancer Center, West China Hospital, Sichuan University, Chengdu, China

**Keywords:** metformin, non-small cell lung cancer, epidermal growth factor receptor tyrosine kinase inhibitor, chemotherapy, immune checkpoint inhibitor, prognosis

## Abstract

**Background and purpose:** Previous studies have found that metformin can inhibit tumor growth and improve outcomes for cancer patients. However, the association between the addition of metformin to the treatment regimen and survival in non-small cell lung cancer (NSCLC) patients receiving antineoplastic agents such as chemotherapy drugs, epidermal growth factor receptor tyrosine kinase inhibitors (EGFR-TKIs), and immune checkpoint inhibitors (ICIs) remains unclear. This study aimed to evaluate the effect of metformin in NSCLC patients who received the aforementioned antineoplastic therapies.

**Methods:** Several electronic databases were searched for relevant studies published by 10 September 2022. The primary and secondary outcomes were overall survival (OS) and progression-free survival (PFS); eligible studies were those comparing patients with and without the addition of metformin. Hazard ratios (HRs) and 95% confidence intervals (CIs) were combined, with all statistical analyses performed using STATA 15.0.

**Results:** A total of 19 studies involving 6,419 participants were included, of which six were randomized controlled trials. The overall pooled results indicate that the addition of metformin improved OS (HR = 0.84, 95% CI: 0.71–0.98, *p* = 0.029) and PFS (HR = 0.85, 95% CI: 0.74–0.99, *p* = 0.039). However, subgroup analysis based on treatment type and comorbidity of diabetes mellitus demonstrated that improvements in OS and PFS were observed only in diabetic and EGFR-TKI-treated patients (OS: HR = 0.64, 95% CI: 0.45–0.90, *p* = 0.011; PFS: HR = 0.59, 95% CI: 0.34–1.03, *p* = 0.061).

**Conclusion:** Overall, this meta-analysis found that metformin use could improve outcomes for diabetic patients receiving EGFR-TKIs. However, no significant association between the addition of metformin and the survival of non-diabetic NSCLC patients receiving chemotherapy or ICI therapy was identified based on the current evidence.

## Introduction

The survival rate of patients with locally advanced or metastatic non-small cell lung cancer (NSCLC) remains poor, with the opportunity for radical resection having been lost by the time of diagnosis for a significant proportion of NSCLC patients (Kang et al., 2022; Xia et al., 2022; Xiang et al., 2022). Chemotherapy, epidermal growth factor receptor tyrosine kinase inhibitors (EGFR-TKIs), and immune checkpoint inhibitors (ICIs) are currently the main treatments for advanced NSCLC patients. However, the overall therapeutic effects of these treatments are not satisfactory, and their clinical use is usually limited for various reasons, such as primary and acquired resistance. Therefore, other medical measures are needed to improve the therapeutic effects of these antineoplastic agents.

Metformin (1,1-dimethylbiguanide) is the most commonly used drug for treating type 2 diabetes mellitus. In recent decades, substantial evidence has suggested that there is a clear beneficial effect of metformin in cases of malignancies, and that metformin plays a significant role in reducing cancer risk and improving outcomes for cancer patients (Mu et al., 2022; Yao et al., 2022; Zhang Q. et al., 2022; Han et al., 2023). A number of studies have found that metformin could reduce cancer morbidity and mortality rates in diabetic patients (Brancher et al., 2021; Kang et al., 2021). The underlying mechanisms are complicated and involve numerous pathways, such as the liver kinase B1 (LKB1)-dependent AMPK pathway and the GRB/IRS-1/PI3K/AKT/mTOR pathway, and the regulation of certain targets, such as the silent information regulator T1 (SIRT1) and YAP (Han et al., 2023).

In the case of lung cancer, a meta-analysis by Xiao et al. (2020) demonstrated that metformin treatment is significantly associated with reduced NSCLC incidence (HR = 0.78, 95% CI: 0.70–0.86). Many studies have verified the beneficial role of metformin in NSCLC patients after surgical resection (Medairos et al., 2016; Yendamuri et al., 2019). However, as mentioned previously, a certain proportion of patients with advanced or inoperable NSCLC receive non-surgical treatments, including chemotherapy, EGFR-TKIs, and ICI therapy, which have become common in recent years. It remains unclear whether the concurrent use of metformin could enhance the efficacy of the aforementioned medications and improve the survival of NSCLC patients.

Luo et al. (2021) conducted a meta-analysis to explore the value of metformin as an adjunct treatment alongside antineoplastic agents in lung cancer; they showed that the addition of metformin might improve survival outcomes for lung cancer patients. However, their results were limited in terms of identifying an association between metformin use and the survival of NSCLC patients receiving the aforementioned antineoplastic agents.

Therefore, the aim of this meta-analysis was to identify the value of the concurrent use of metformin during treatment of NSCLC patients with chemotherapy, EGFR-TKIs, and ICIs. The results might contribute to the clinical application of metformin in NSCLC patients receiving the aforementioned antineoplastic agents.

## Materials and methods

This meta-analysis was conducted according to the Preferred Reporting Items for Systematic Reviews and Meta-Analyses guidelines (2020) (Zhang et al., 2020).

### Literature search

The PubMed, Embase, and Web of Science electronic databases were searched for articles published in the period from their inception to 10 September 2022. The following keywords were used during literature retrieval: metformin, lung, pulmonary, tumor, cancer, carcinoma, neoplasm, survival, prognosis, and prognostic. To avoid omissions, a relatively broad search strategy was developed and implemented, using the expression: metformin AND (lung OR pulmonary) AND (tumor OR cancer OR carcinoma OR neoplasm) AND (survival OR prognosis OR prognostic). In addition, MeSH terms and free words were applied, and references cited in the included publications were also reviewed.

### Inclusion and exclusion criteria

The inclusion criteria were as follows: 1) patients were pathologically diagnosed with primary NSCLC and received chemotherapy, EGFR-TKIs, or ICI therapy; 2) overall survival (OS) and/or progression-free survival (PFS) were compared between patients who did and did not also receive metformin during treatment with the aforementioned therapies; 3) hazard ratios (HRs) with 95% confidence intervals (CIs) were directly reported by the article.

The exclusion criteria were as follows: 1) letters, editorials, meeting abstracts, case reports, and reviews; 2) articles reporting insufficient, overlapping, or duplicated data.

### Data extraction

The following information was collected from each included study: the name of the first author; publication year; country; sample size; study design, including randomized controlled trials (RCTs) and cohort studies; tumor–node–metastasis (TNM) stage; treatment strategy; comorbidity of diabetes mellitus; endpoint; and HR and 95% CI.

### Methodological quality assessment

The quality of the RCTs and cohort studies was assessed using the Jadad scale and the Newcastle–Ottawa scale (NOS), respectively (Bhandari et al., 2001; Wang J. L. et al., 2021). Studies with a Jadad score of 4 or a NOS score of 6 or higher were defined as high-quality studies.

The literature search, selection, data collection, and quality assessment were all performed by two authors independently, and all disagreements were resolved by team discussion.

### Statistical analysis

All statistical analyses carried out in this meta-analysis were conducted using STATA 15.0 software. HRs with 95% CIs were combined to compare OS and PFS between patients who did and did not receive metformin. Heterogeneity among the included studies was evaluated via I^2^ statistics and Q tests. When significant heterogeneity was observed, in the form of I^2^ > 50% or *p* < 0.1, a random-effects model was applied; otherwise, a fixed-effects model was used (Barili et al., 2018). Subgroup analysis, stratified by treatment type, comorbidity of diabetes mellitus, and study design, was additionally conducted. In addition, a sensitivity analysis was conducted to identify the sources of heterogeneity and evaluate the stability of the pooled results. Furthermore, Begg’s funnel plots were constructed and Egger’s tests were conducted to detect publication bias (Begg and Mazumdar, 1994; Egger et al., 1997). Significant publication bias was defined as *p* < 0.05.

## Results

### Literature search and retrieval

**FIGURE 1:**
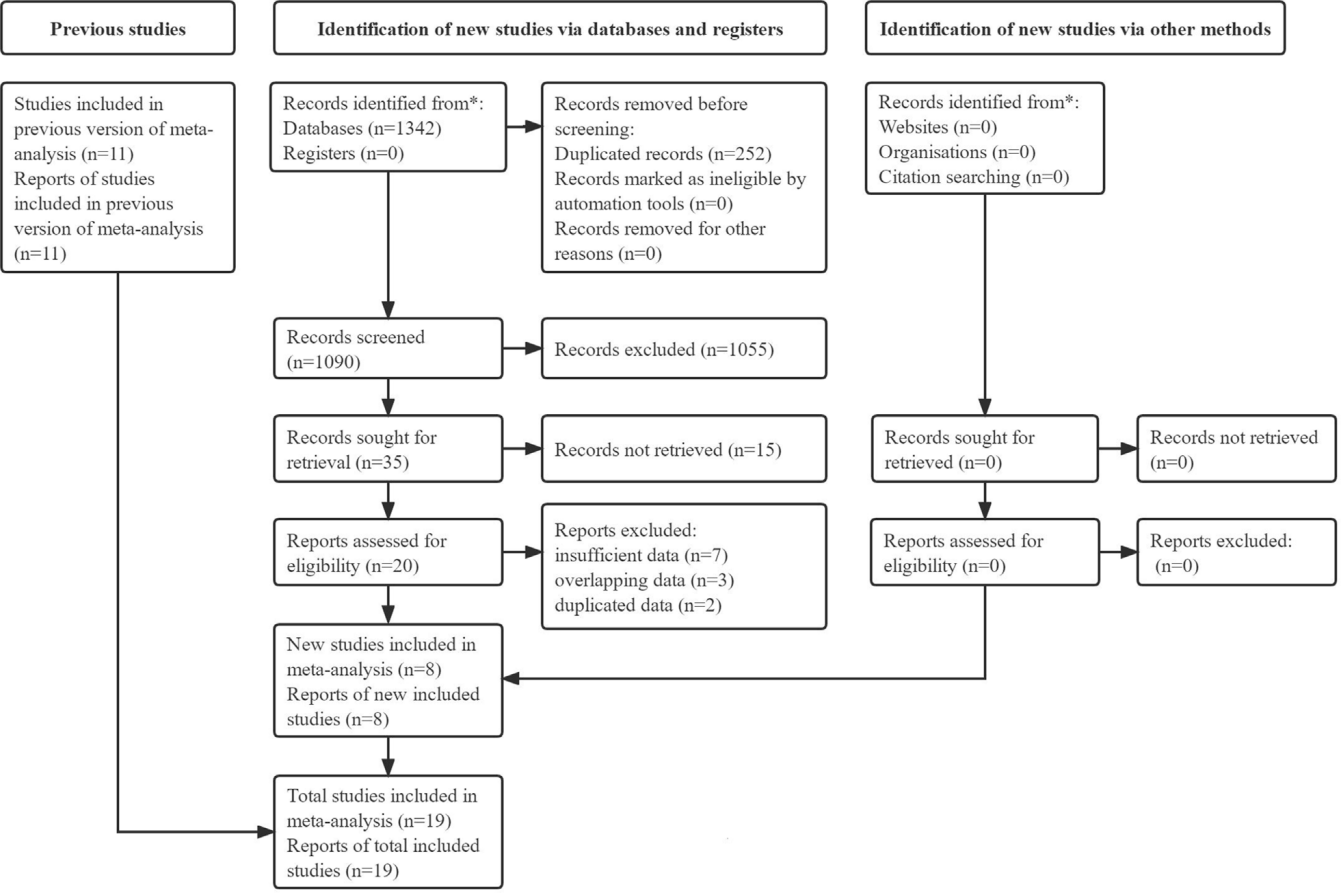
Flowchart of literature retrieval and inclusion in this meta-analysis.

Initially, 1,342 records were identified from electronic databases, and 252 duplicated records were removed. Next, 1,055 irrelevant publications and 15 unavailable publications were excluded after review of the titles and abstracts, respectively. Eight of the remaining reports were included after review of the full texts, and 11 available studies were included from previous relevant meta-analyses. Thus, a total of 19 studies were included in this meta-analysis (Tan et al., 2011; Ahmed et al., 2015; Chen et al., 2015; Lin et al., 2015; Sayed et al., 2015; Wink et al., 2016; Wen-Xiu et al., 2018; Afzal et al., 2019; Arrieta et al., 2019; Hung et al., 2019; Li et al., 2019; Su et al., 2020; Cortellini et al., 2021; Han et al., 2021; Jacobi et al., 2021; Lee et al., 2021; Skinner et al., 2021; Tsakiridis et al., 2021; Wang Y. et al., 2021). The specific literature retrieval process is presented in Figure 1.

### Basic characteristics of the included studies

The included studies were published between 2011 and 2021, and their sample sizes ranged from 40 to 1,633, for a total of 6,419 patients. Six of the studies were RCTs (Sayed et al., 2015; Arrieta et al., 2019; Li et al., 2019; Lee et al., 2021; Skinner et al., 2021; Tsakiridis et al., 2021); the remainder were cohort studies (Tan et al., 2011; Ahmed et al., 2015; Chen et al., 2015; Lin et al., 2015; Wink et al., 2016; Wen-Xiu et al., 2018; Afzal et al., 2019; Hung et al., 2019; Su et al., 2020; Cortellini et al., 2021; Han et al., 2021; Jacobi et al., 2021; Wang Y. et al., 2021). All included studies were high-quality studies with a Jadad score ≥4 or NOS score ≥6. Detailed information is presented in Table 1.

**TABLE 1:** Basic characteristics of included studies.

Author	Country	Sample size	Study design	TNM stage	Treatment	Diabetes mellitus	Endpoint	Jadad/NOS score
Tan et al. (2011)	China	99	Cohort	II–IV	CT	Yes	OS, PFS	9
Ahmed et al. (2015)	United States	40	Cohort	I–IV	CRT	Yes	OS, PFS	7
Chen et al. (2015)	China	90	Cohort	III–IV	EGFR-TKI	Yes	OS, PFS	7
Lin et al. (2015)	United States	340	Cohort	IV	CT	Yes	OS	8
Sayed et al. (2015)	Egypt	30	RCT	IV	CT	No	OS, PFS	5
Wink et al. (2016)	Netherlands	682	Cohort	II–III	CRT	Yes	OS, PFS	8
Wen-Xiu et al. (2018)	China	75	Cohort	I-IV	CT	Yes	OS	9
Afzal et al. (2019)	United States	50	Cohort	IV	ICI	Mixed	OS, PFS	8
Arrieta et al. (2019)	Mexico	139	RCT	III–IV	EGFR-TKI	No	OS, PFS	4
Hung et al. (2019)	China	1,633	Cohort	III–IV	EGFR-TKI	Yes	OS, PFS	8
Li et al. (2019)	China	224	RCT	III–IV	EGFR-TKI	No	OS, PFS	7
Su et al. (2020)	China	853	Cohort	IIIB–IV	EGFR-TKI	NR	OS	7
Cortellini et al. (2021)	Italy	950	Cohort	IV	ICI	NR	OS, PFS	6
Han et al. (2021)	China	85	Cohort	I–IV	EGFR-TKI	Yes	OS, PFS	9
Jacobi et al. (2021)	Israel	249	Cohort	IV	ICI	Yes	OS, PFS	8
Lee et al. (2021)	South Korea	164	RCT	IIIB–IV	CT	Mixed	OS, PFS	5
Skinner et al. (2021)	Canada	167	RCT	III	CRT	No	OS, PFS	5
Tsakiridis et al. (2021)	Canada	54	RCT	III	CRT	No	OS, PFS	4
Wang J. L. et al. (2021)	China	495	Cohort	Advanced	CT	Yes	OS	8

RCT: randomized controlled trial; TNM: tumor–node–metastasis; CT: chemotherapy; CRT: chemoradiotherapy; ICI: immune checkpoint inhibitor; EGFR-TKI: epidermal growth factor receptor-tyrosine kinase inhibitor; NR: not reported; OS: overall survival; PFS: progression-free survival; NOS: Newcastle–Ottawa Scale.

### The association between addition of metformin and OS of NSCLC patients receiving antineoplastic agents

**FIGURE 2:**
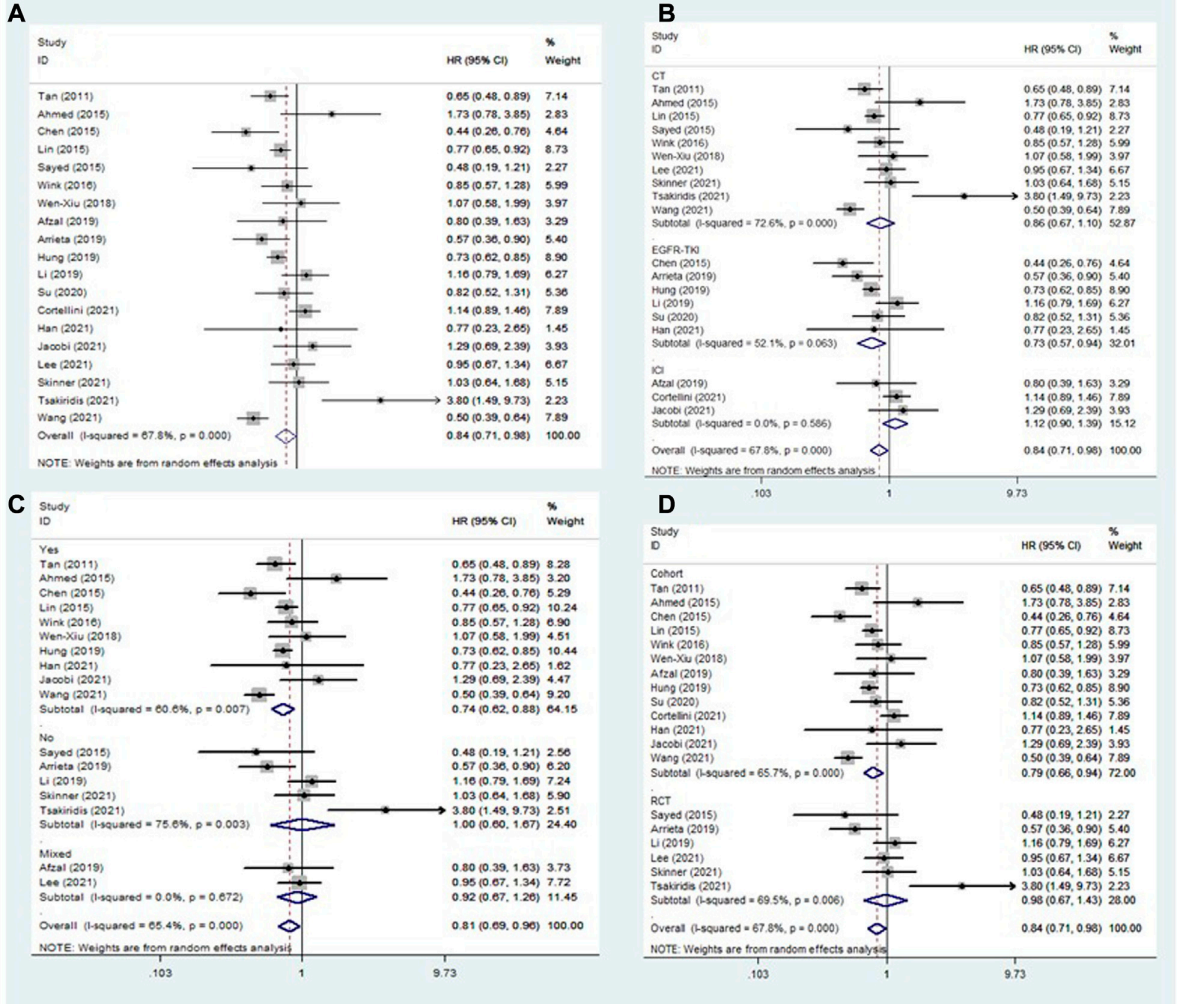
The association between metformin use and overall survival in **(A)** all NSCLC patients; **(B)** subgroups by treatment type; **(C)** subgroups by comorbidity of diabetes mellitus; and **(D)** subgroups by study design. NSCLC: non-small cell lung cancer.

All included studies explored the association between the addition of metformin and the OS of NSCLC patients receiving antineoplastic agents (Afzal et al., 2019; Ahmed et al., 2015; Arrieta et al., 2019; Chen et al., 2015; Cortellini et al., 2021; Han et al., 2021; Hung et al., 2019; Jacobi et al., 2021; Lee et al., 2021; Li et al., 2019; J. J; Lin et al., 2015; Sayed et al., 2015; Skinner et al., 2021; Su et al., 2020; Tan et al., 2011; Tsakiridis et al., 2021; Wang J. L. et al., 2021; Wen-Xiu et al., 2018; Wink et al., 2016). The overall pooled results indicated that patients who were treated with metformin had better rates of OS (HR = 0.84, 95% CI: 0.71–0.98, *p* = 0.029; I^2^ = 67.8%, *p* < 0.001) (Figure 2A). However, subgroup analysis based on treatment type and comorbidity of diabetes mellitus showed that the effect of improved OS was observed only for patients receiving EGFR-TKIs (HR = 0.73, 95% CI: 0.57–0.94, *p* = 0.013) (Figure 2B) and those with diabetes mellitus (HR = 0.74, 95% CI: 0.62–0.88, *p* = 0.001) (Figure 2C). In contrast, the association between metformin use and the OS of NSCLC patients receiving chemoradiotherapy was negative (HR = 1.36, 95% CI: 0.79–2.35, *p* = 0.261). In addition, subgroup analysis based on study design showed that the association between metformin use and improved OS in NSCLC patients was observed only in cohort studies (HR = 0.79, 95% CI: 0.66–0.94, *p* = 0.007) (Figure 2D).

To further clarify the nature of the association between metformin use and OS in EGFR-TKI-treated and diabetic NSCLC patients, subgroup analyses were conducted focusing on EGFR-TKI-treated patients (according to comorbidity of diabetes mellitus) and diabetic patients (according to treatment type). The pooled results demonstrated that the addition of metformin could significantly improve the OS of diabetic NSCLC patients receiving EGFR-TKIs (HR = 0.64, 95% CI: 0.45–0.90, *p* = 0.011) (Table 2).

**TABLE 2:** Results of meta-analysis.

	No. of studies	HR	95% CI	*p*-value	I^2^ (%)	P_heterogeneity_
Overall survival	19	0.84	0.71–0.98	0.029	67.8	<0.001
Treatment						
Chemotherapy	10	0.86	0.67–1.10	0.230	72.6	<0.001
With radiotherapy	4	1.36	0.79–2.35	0.261	68.6	0.023
EGFR-TKI	6	0.73	0.57–0.94	0.013	52.1	0.063
With diabetes mellitus	3	0.64	0.45–0.90	0.011	37.0	0.205
Without diabetes mellitus	2	0.82	0.41–1.65	0.583	81.7	0.019
Immune checkpoint inhibitor	3	1.12	0.90–1.39	0.311	0.0	0.586
Comorbidity of diabetes mellitus						
Yes	10	0.74	0.62–0.88	0.001	60.6	0.007
Chemotherapy	6	0.76	0.59–0.97	0.030	68.9	0.007
EGFR-TKI	3	0.64	0.45–0.90	0.011	37.0	0.205
Immune checkpoint inhibitor	1	1.29	0.69–2.40	0.422	-	-
No	5	1.00	0.60–1.67	0.998	75.6	0.003
Mixed	2	0.92	0.67–1.26	0.598	0.0	0.672
Study design						
Cohort study	13	0.79	0.66–0.94	0.007	65.7	<0.001
Randomized controlled trial	6	0.98	0.67–1.43	0.910	69.5	0.006
Progression-free survival	15	0.85	0.74–0.99	0.039	60.2	0.001
Treatment						
Chemotherapy	7	0.94	0.71–1.24	0.643	66.4	0.007
With radiotherapy	4	1.18	0.69–2.00	0.551	72.8	0.011
EGFR-TKI	5	0.70	0.52–0.94	0.019	71.3	0.008
With diabetes mellitus	3	0.59	0.34–1.03	0.061	80.3	0.006
Without diabetes mellitus	2	0.81	0.48–1.37	0.431	73.4	0.053
Immune checkpoint inhibitor	3	1.02	0.83–1.24	0.864	0.0	0.859
Comorbidity of diabetes mellitus						
Yes	7	0.75	0.61–0.92	0.006	60.5	0.019
Chemotherapy	3	0.77	0.59–1.02	0.070	37.8	0.201
EGFR-TKI	3	0.59	0.34–1.03	0.061	80.3	0.006
Immune checkpoint inhibitor	1	1.08	0.61–1.92	0.793	-	-
No	5	0.96	0.64–1.44	0.839	71.8	0.007
Mixed	2	0.98	0.72–1.31	0.868	0.0	0.676
Study design						
Cohort study	9	0.80	0.68–0.95	0.011	57.5	0.016
Randomized controlled trial	6	0.97	0.71–1.32	0.828	65.0	0.014

HR: hazard ratio; CI: confidence interval; EGFR-TKI: epidermal growth factor receptor-tyrosine kinase inhibitor.

### The association between addition of metformin and PFS of NSCLC patients receiving antineoplastic agents

**FIGURE 3:**
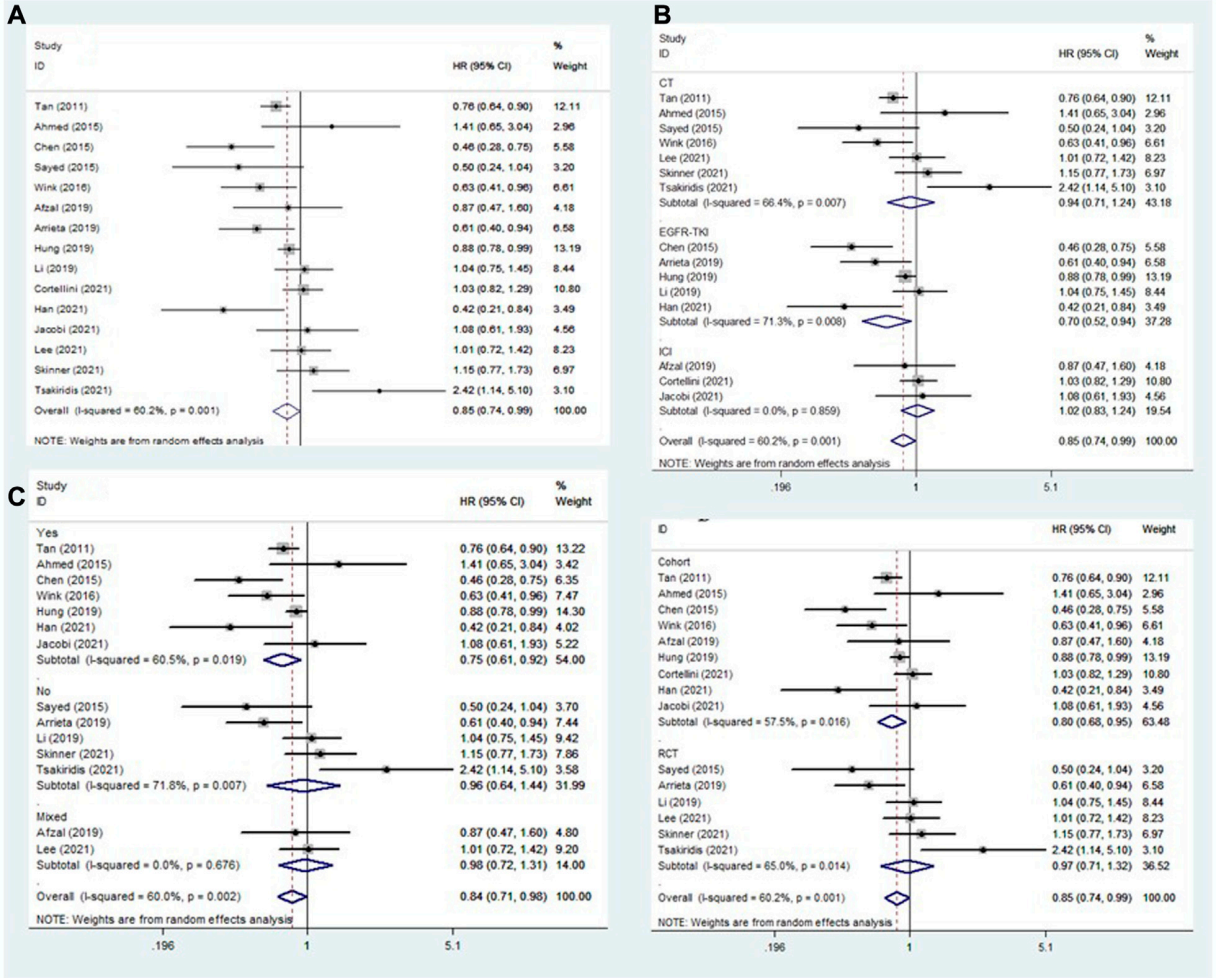
The association between metformin use and progression-free survival in **(A)** all NSCLC patients; **(B)** subgroups by treatment type; **(C)** subgroups by comorbidity of diabetes mellitus; and **(D)** subgroups by study design. NSCLC: non-small cell lung cancer.

Fifteen studies explored the association between the addition of metformin and PFS in NSCLC patients receiving antineoplastic agents (Tan et al., 2011; Ahmed et al., 2015; Chen et al., 2015; Sayed et al., 2015; Wink et al., 2016; Afzal et al., 2019; Arrieta et al., 2019; Hung et al., 2019; Li et al., 2019; Cortellini et al., 2021; Han et al., 2021; Jacobi et al., 2021; Lee et al., 2021; Skinner et al., 2021; Tsakiridis et al., 2021). The overall results showed that metformin use was clearly related to better rates of PFS (HR = 0.85, 95% CI: 0.74–0.99, *p* = 0.039; I^2^ = 60.2%, *p* = 0.001) (Figure 3A). However, subgroup analysis stratified by treatment type and comorbidity of diabetes mellitus also indicated that the effect of improved PFS was observed only in patients receiving EGFR-TKIs (HR = 0.70, 95% CI: 0.52–0.94, *p* = 0.019) (Figure 3B) and those with diabetes mellitus (HR = 0.75, 95% CI: 0.61–0.92, *p* = 0.006) (Figure 3C). In contrast, the association between metformin use and PFS of NSCLC patients receiving chemoradiotherapy was negative (HR = 1.18, 95% CI: 0.69–2.00, *p* = 0.551). In addition, subgroup analysis based on study design showed that the association between metformin use and improved PFS in patients with NSCLC was observed only in cohort studies (HR = 0.80, 95% CI: 0.68–0.95, *p* = 0.011) (Figure 3D).

Similarly, subgroup analyses were performed focusing on patients receiving EGFR-TKI treatment (according to comorbidity of diabetes mellitus) and diabetic patients (according to treatment type). The pooled results demonstrated that the addition of metformin was related to better PFS in diabetic NSCLC patients receiving EGFR-TKIs (HR = 0.59, 95% CI: 0.34–1.03, *p* = 0.061), although the difference was not significant (Table 2).

### Sensitivity analysis

**FIGURE 4:**
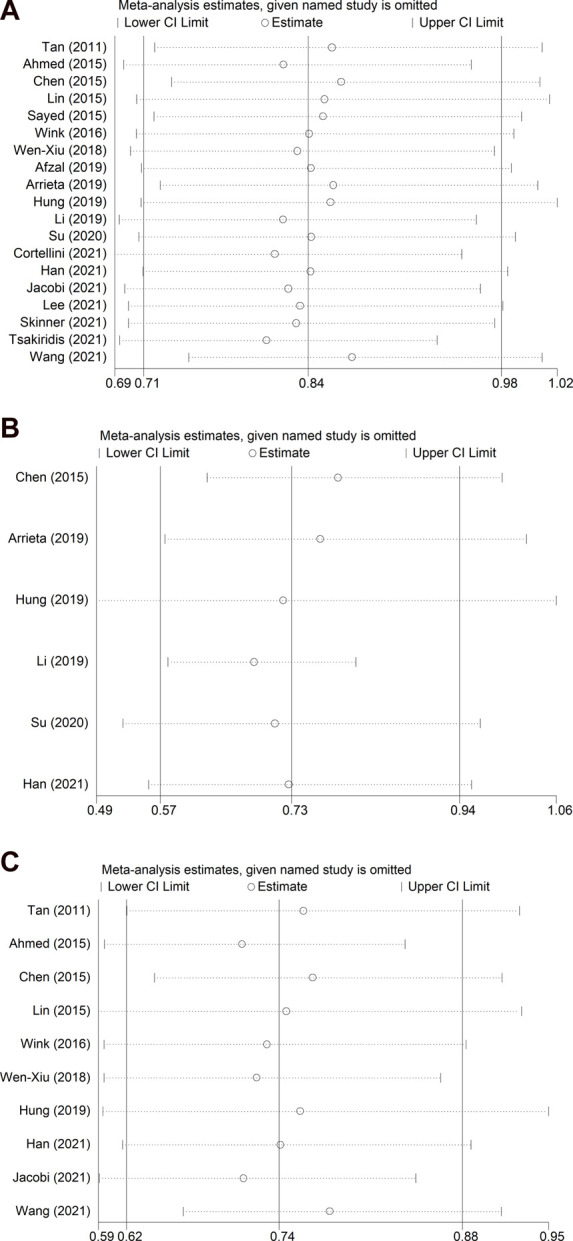
Sensitivity analysis for the association between metformin use and overall survival in **(A)** all NSCLC patients; **(B)** patients receiving EGFR-TKI therapy; and **(C)** patients with diabetes mellitus. NSCLC: non-small cell lung cancer; EGFR-TKI: epidermal growth factor receptor-tyrosine kinase inhibitor.

**FIGURE 5:**
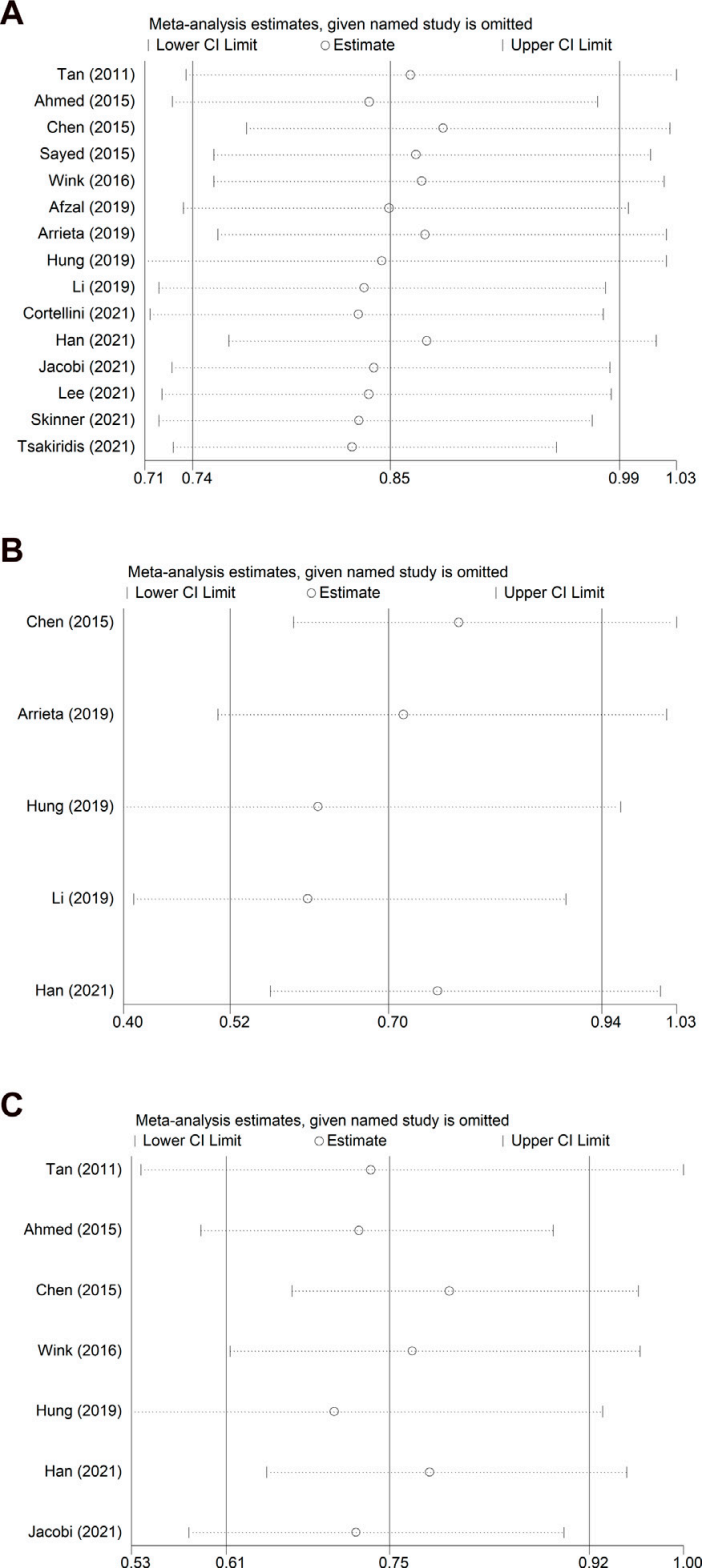
Sensitivity analysis for the association between metformin use and progression-free survival in **(A)** all NSCLC patients; **(B)** patients receiving EGFR-TKI therapy; and **(C)** patients with diabetes mellitus. NSCLC: non-small cell lung cancer; EGFR-TKI: epidermal growth factor receptor-tyrosine kinase inhibitor.

Sensitivity analyses were conducted for OS and PFS, focusing on all NSCLC patients (Figures 4A, 5A), EGFR-TKI-treated patients (Figures 4B, 5B), and diabetic patients (Figures 4C, 5C). Overall, a small number of the included studies had a clear impact on the results. More RCTs with large samples are needed to verify our findings.

### Publication bias

**FIGURE 6:**
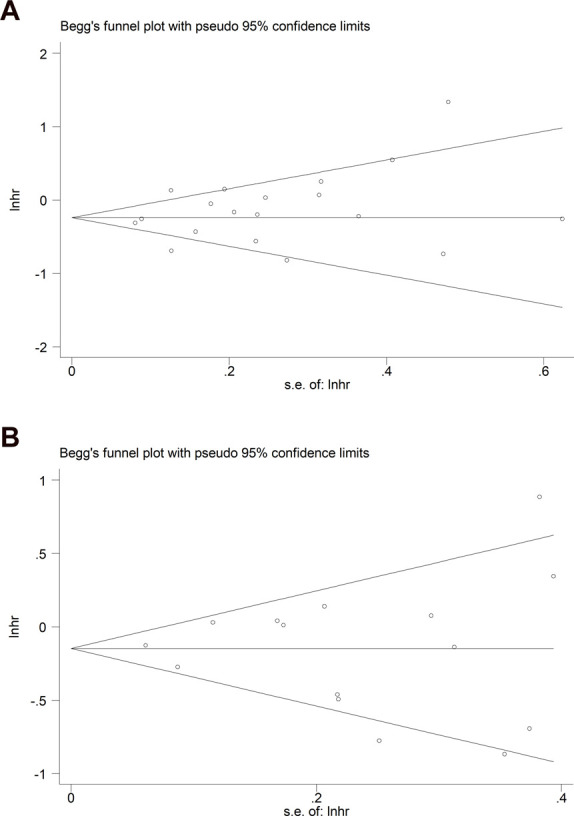
Begg’s funnel plots for **(A)** overall survival and **(B)** progression-free survival.

Begg’s funnel plots for OS and PFS were both symmetrical (Figures 6A, B), and the *p*-values in Egger’s tests for OS and PFS were 0.218 and 0.900, respectively, indicating that no significant publication bias existed in this meta-analysis.

## Discussion

The current meta-analysis demonstrated that the addition of metformin was beneficial for diabetic NSCLC patients who received EGFR-TKI therapy and that metformin use could significantly improve the survival rate of this group of patients. However, no significant association between the addition of metformin and the survival of non-diabetic patients receiving chemotherapy or ICI was observed. Furthermore, due to the limitations of the included studies, more RCTs with larger samples are needed to verify the beneficial value of metformin use in diabetic and EGFR-TKI-treated NSCLC patients.

In a previous similar meta-analysis, Luo et al. (2021) included three RCTs and 11 observational cohort studies involving 3,856 lung cancer patients, and showed that antineoplastic agents combined with metformin significantly improve OS (HR = 0.73, *p* < 0.001) and PFS (HR = 0.72, *p* = 0.001). Similar results were observed when they combined cohort studies, but no significant association between metformin use and survival of lung cancer patients was detected based on limited data from RCTs. The authors conducted additional subgroup analyses based on type of therapy (chemotherapy vs EGFR-TKI), histology (NSCLC vs small cell lung cancer), and stage (III–IV vs I–IV), which produced consistent results, indicating that the addition of metformin could induce clear improvement in the efficacy of antineoplastic agents in lung cancer patients (Luo et al., 2021). However, this conclusion is obviously crude, and several factors—such as therapeutic methods and histology—may affect the objectivity and authenticity of the results.

Similarly, several other meta-analyses have explored the anticancer effect of metformin in cases of lung cancer in recent years, but there are considerable limitations to these analyses (Zhang Q. et al., 2022). Zhang F. et al. (2022) explored the anticancer role of metformin in NSCLC patients receiving EGFR-TKIs, but only three studies published before August 2020 were included. Brancher et al. (2021) included ten cohort studies and four RCTs and came to the tentative conclusion that metformin use might be associated with improved OS of lung cancer patients; this result was similar to that of several other meta-analyses (Wan et al., 2016; Cao et al., 2017; Zhong et al., 2017; Zhang et al., 2018; Zeng et al., 2019; Xiao et al., 2020; Brancher et al., 2021). Thus, we conducted the current meta-analysis to clarify the value of metformin use in NSCLC patients receiving the aforementioned antineoplastic agents. We demonstrated that benefits of metformin were only experienced by diabetic and EGFR-TKI-treated NSCLC patients.

Several studies have investigated the underlying mechanisms by which metformin affects the therapeutic effects of EGFR-TKIs in NSCLC patients. It has been reported that metformin and EGFR-TKIs have a synergistic therapeutic effect in NSCLC patients with type 2 diabetes (Nguyen et al., 2011). In one study focusing on the treatment of LKB1 wild-type NSCLC cells, it was found that the addition of gefitinib to metformin could inhibit EGFR phosphorylation and its downstream signaling; in addition, increased c-Raf/B-Raf isomerization was found to cause MAPK activation, which induced significant apoptosis *in vitro* and *in vivo* (Morgillo et al., 2013). Furthermore, metformin plays a role in overcoming resistance to EGFR-TKIs by inhibiting the PI3K/AKT/mTOR signaling pathway (Tan, 2020). Li et al. (2014) also reported on the effects of metformin in inhibiting the IL-6/STAT3 signaling pathway, reversing epithelial–mesenchymal transition (EMT), and overcoming EGFR-TKI drug (gefitinib and erlotinib) resistance in NSCLC cells. However, most relevant studies have been conducted *in vitro* and *in vivo*, and the mechanisms are still unclear.

Although we found that metformin was not significantly associated with the survival of NSCLC patients receiving chemotherapy drugs, a number of studies have indicated a role for metformin in increasing the sensitivity of chemotherapy drugs such as doxorubicin, cisplatin, and paclitaxel in NSCLC (Iliopoulos et al., 2011; Tan et al., 2011; Teixeira et al., 2013; Tseng et al., 2013). For example, cisplatin resistance is associated with signal transducer and activator of transcription 3 (STAT3) phosphorylation, production of reactive oxygen species (ROS), and IL-6 secretion, but metformin could inhibit cisplatin-induced STAT3 phosphorylation (through the LKB1-AMPK and mTOR pathway-dependent mechanisms), ROS generation, and autocrine IL-6 secretion (Khan et al., 2013; Lin et al., 2013). Metformin thus plays a role in enhancing cisplatin cytotoxicity and improving the cisplatin resistance of cancer cells (Wang et al., 2014; Wang et al., 2015).

No significant relationship between metformin use and the survival of ICI-treated NSCLC patients was observed in our meta-analysis. A few previous studies have suggested that metformin enhances the efficacy of ICI therapy in NSCLC patients (Yendamuri et al., 2019; Shen et al., 2020; Kim et al., 2021). The expression levels of LKB1 and PD-L1 are closely correlated, and AMPK inhibition reduces PD-L1 levels in NSCLC cells through LKB1 (Shen et al., 2020). Metformin has the ability to enhance the expression of LKB1 and the activation of AMPK, which improves the therapeutic effect of ICIs in NSCLC (Shen et al., 2020). However, most relevant studies investigating the therapeutic role of metformin in NSCLC patients receiving chemotherapy and ICIs have been based on cellular and animal trials. Therefore, more prospective RCTs are needed to clarify the therapeutic role of metformin in NSCLC patients receiving the aforementioned therapies.

There are several limitations to our meta-analysis. First, most of the studies included were retrospective cohort studies with small sample sizes, which might cause some bias. Second, although stratification analysis was performed based specifically on treatment type (EGFR-TKI therapy) and comorbidity of diabetes mellitus and strongly indicated a significant relationship between metformin use and better outcomes for diabetic and EGFR-TKI-treated NSCLC patients, only 1808 patients from three cohort studies were enrolled (Chen et al., 2015; Hung et al., 2019; Han et al., 2021) in this group. Third, due to a lack of original data, we were unable to conduct additional subgroup analyses based on other important parameters, such as metformin dose, TNM stage, and age. Fourth, we were unable to calculate the base and recommended doses of metformin to determine its effect in enhancing the efficacy of EGFR-TKIs in this meta-analysis. Five clear instances of heterogeneity among the included studies were observed in the analysis; however, unfortunately, the sources of heterogeneity were not clarified in the subgroup analyses, and due to the limited amount of evidence, we were unable to conduct more subgroup analyses based on other parameters.

## Conclusion

The addition of metformin to the treatment regimen was beneficial for diabetic NSCLC patients who received EGFR-TKI therapy, and metformin use could significantly improve the survival rate of this group of patients. However, no significant association between the addition of metformin and the survival of non-diabetic NSCLC patients receiving chemotherapy or ICI therapy was identified based on the current evidence. Meanwhile, more RCTs with larger samples are needed to verify the aforementioned findings.

## Data Availability

The original contributions presented in the study are included in the article/Supplementary Material, further inquiries can be directed to the corresponding authors.
